# Supplementary material for: Degradation of Dioxins and DBF in Urban Soil Microcosms from Lausanne (Switzerland): Functional Performance of Indigenous Bacterial Strains
Source: Microorganisms. 2025 Oct 5;13(10):2306. doi: 10.3390/microorganisms13102306 (PMC12566063; doi:10.3390/microorganisms13102306)
Supplement: Supplementary file 1 [file microorganisms-13-02306-s001.zip › microorganisms-3886203-supplementary.pdf]

Supplementary information

Table S1

Dioxin and furan concentrations measured in soil samples collected from three urban sites in Lausanne, Switzerland: Eracom (ERA), Signal (SIG), and Ancien Stand (AST). Coordinates are expressed in decimal degrees (WGS84). Concentrations of individual congeners are reported in ng/kg of dry soil. The table includes polychlorinated dibenzo-p-dioxins (PCDDs), polychlorinated dibenzofurans (PCDFs), and octachlorinated congeners (OCDD and OCDF). Toxic equivalent values (TEQ) were calculated according to WHO-2005 toxic equivalency factors and are presented under three quantification assumptions: including 0%, 50%, or 100% of the lower-bound quantification limit (LQ).

	**Eracom**	**Signal**	**Ancien-Stand**
Coordinates	**46.5223° N, 6.6226° E**	**46.5273° N, 6.6363° E**	**46.5347° N, 6.6289° E**
Abbreviation	**ERA**	**SIG**	**AST**
Compound	**Concentration (ng/kg of soil)**	**Concentration (ng/kg of soil)**	**Concentration (ng/kg of soil)**
1.2.3.4.6.7.8-HpCDD	135.3	484.5	83.8
1.2.3.4.6.7.8-HpCDF	94.8	446.8	74.3
1.2.3.4.7.8.9-HpCDF	6.0	28.0	4.3
1.2.3.4.7.8-HxCDD	11.5	50.0	8.0
1.2.3.4.7.8-HxCDF	18.5	73.0	12.0
1.2.3.6.7.8-HxCDD	13.3	56.5	8.5
1.2.3.6.7.8-HxCDF	18.0	72.0	11.3
1.2.3.7.8.9-HxCDD	15.3	67.5	10.5
1.2.3.7.8-PeCDD	7.5	37.0	6.0
1.2.3.7.8-PeCDF	6.8	30.5	4.8
2.3.4.6.7.8-HxCDF	16.5	69.3	11.8
2.3.4.7.8-PeCDF	8.8	34.5	5.0
2.3.7.8-TCDF	3.0	10.3	2.0
OCDD	248.3	737.0	142.8
OCDF	29.3	150.5	22.8
TEQ (WHO-05) incl 0% LQ	22.4	102.5	15.7
TEQ (WHO-05) incl 100% LQ	23.5	102.5	16.8
TEQ (WHO-05) incl 50% LQ	22.9	102.5	16.3

HpCDD: heptachlorodibenzo-p-dioxin; HpCDF: heptachlorodibenzofuran; HxCDD: hexachlorodibenzo-p-dioxin; HxCDF: hexachlorodibenzofuran; PeCDD: pentachlorodibenzo-p-dioxin; PeCDF: pentachlorodibenzofuran; TCDF: tetrachlorodibenzofuran; OCDD: octachlorodibenzo-p-dioxin; OCDF: octachlorodibenzofuran.

Table S2

GC-MS parameters used for the quantification of the two model compounds: dibenzofuran (DBF) and 2,7-dichlorodibenzo-p-dioxin (2,7-DD). The table reports the selected ion transitions (m/z), retention times (min), and analytical limits, including the limit of detection (LOD) and limit of quantification (LOQ), used in single ion monitoring (SIM) mode. Analyses were performed using a GC-MS system equipped with an OPTIMA-5 MS column under the conditions detailed in the Materials and Methods section.

**Compound**	**Transition m/z**	**Retention time**	**LOD**	**LOQ**
Dibenzofuran	168→ 139			
2,7-dichlorodibenzo-p-dioxin	252→ 126			

Table S3

Antibiotic sensitivity of selected bacterial strains expressed as minimum inhibitory concentrations (MIC, µg/mL), determined via the E-test method. "R" indicates resistance (growth at the highest tested concentration).

Antibiotic	Class	Mode of Action	*P. protegens*	*A. bohemicus*	*P. chlororaphis*	*B. velezensis*	*P. kermanshahensis*
ACC	Beta-lactam	Bactericidal	R	16	R	0.016	0.016
AM	Penicillin	Bactericidal	R	4	R	1	32
CI	Fluoroquinolone	Bactericidal	0.125	0.047	0.047	0.094	0.125
CM	Macrolide	Bactericidal	R	24	R	0.19	R
EM	Macrolide	Bactericidal	R	2	32	0.094	96
GM	Aminoglycoside	Bactericidal	2	0.75	3	0.094	1.5
IP	Carbapenem	Bactericidal	2	0.19	2	0.012	0.19
LE	Fluoroquinolone	Bactericidal	0.75	0.125	0.25	0.064	0.5
LZ	Oxazolidinone	Bacteriostatic	R	128	R	0.75	R
MP	Carbapenem	Bactericidal	R	0.25	4	0.032	0.38
PM	Cephalosporin	Bactericidal	3	1.5	3	0.75	1.5
TC	Tetracycline	Bacteriostatic	12	6	12	4	3
TZ	Cephalosporin	Bactericidal	8	6	2	1.5	1.5
VA	Glycopeptide	Bactericidal	R	96	R	0.25	R

ACC: Clavulanic acid; AM: Ampicillin; CI: Ciprofloxacin; CM: Clindamycin; EM: Erythromycin; GM: Gentamicin; IP: Imipenem; LE: Levofloxacin; LZ: Linezolid; MP: Meropenem; PM: Cefepime; TC: Tetracycline; TZ: Ceftazidime; VA: Vancomycin.

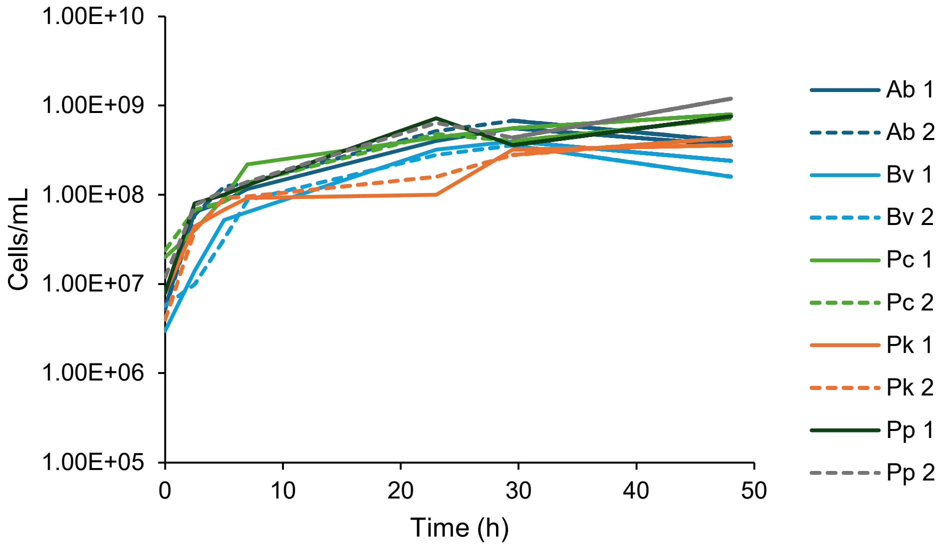

Figure S1

Growth kinetics of isolated bacterial strains during scale-up in TSB. Cell concentrations (cells/mL) were measured over a 50-hour incubation period at 25 °C. Under these generic conditions, all strains reached high cell densities (10^8–10^9 cells/mL) within 24 hours, demonstrating efficient growth in non-selective medium. Each strain was tested in duplicate (1 and 2 represent independent parallel replicates). Ab: *Acinetobacter bohemicus*; Bv: *Bacillus velezensis*; Pc: *Pseudomonas chlororaphis*; Pk: *Pseudomonas kermanshahensis*; Pp: *Pseudomonas protegens*.

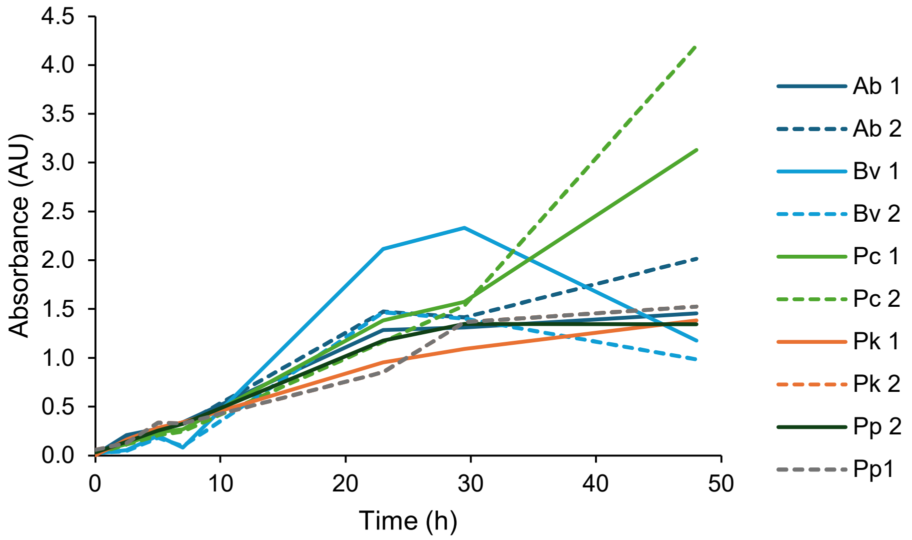

Figure S2

Optical density (OD600) measurements of bacterial cultures during scale-up in TSB. OD600 was monitored over a 50-hour incubation period at 25 °C. All strains showed increasing turbidity, indicative of growth. However, *Bacillus velezensis* (Bv) and *Pseudomonas chlororaphis* (Pc) displayed elevated absorbance after 30 hours, likely due to the production of light-absorbing metabolites (e.g., pigments or proteins). Each strain was tested in duplicate (1 and 2 represent independent parallel replicates). Ab: *Acinetobacter bohemicus*; Bv: *Bacillus velezensis*; Pc: *Pseudomonas chlororaphis*; Pk: *Pseudomonas kermanshahensis*; Pp: *Pseudomonas protegens*.

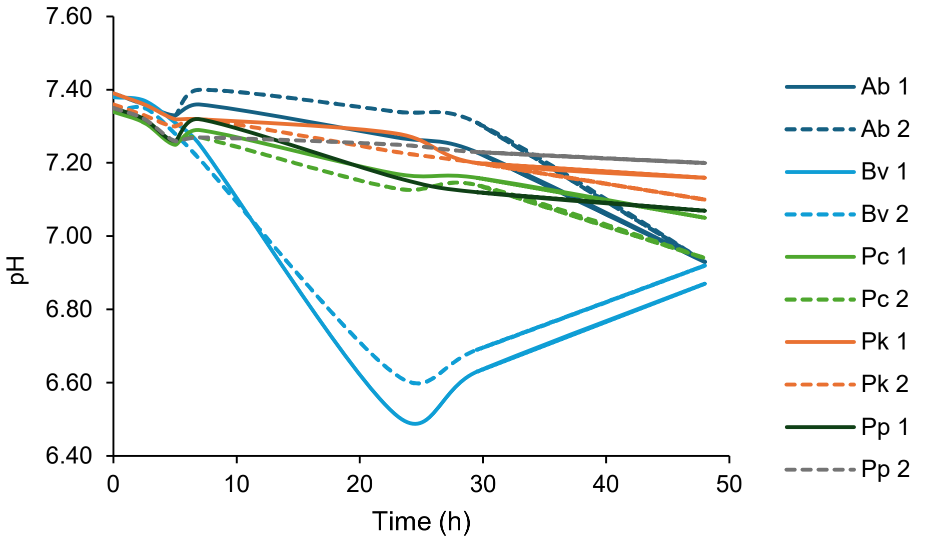

Figure S3

Variation in culture pH during bacterial scale-up in 2 L TSB medium over 50 h. All strains maintained stable metabolic activity, with pH values fluctuating within a narrow range (6.5–7.4). Each strain was tested in duplicate (1 and 2 represent independent parallel replicates). Ab: *Acinetobacter bohemicus*; Bv: *Bacillus velezensis*; Pc: *Pseudomonas chlororaphis*; Pk: *Pseudomonas kermanshahensis*; Pp: *Pseudomonas protegens*.

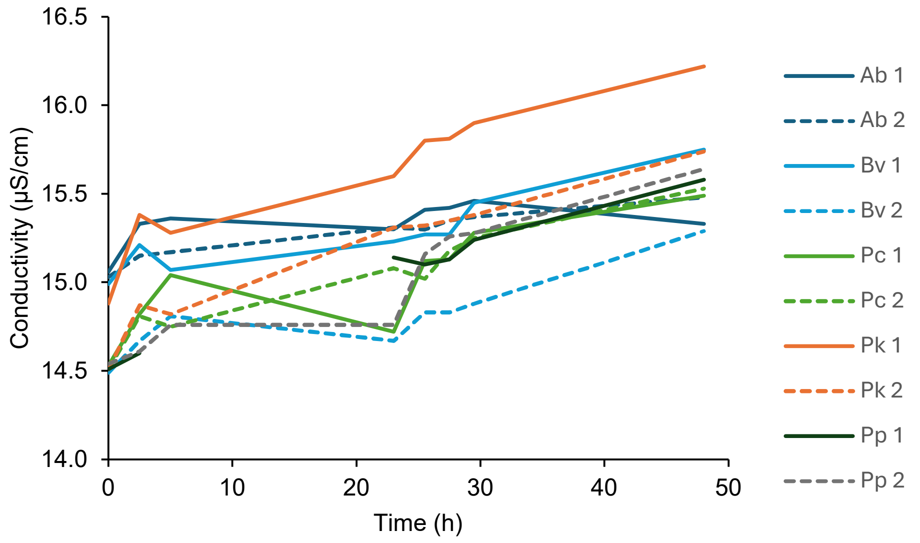

Figure S4

Conductivity profiles of bacterial cultures during scale-up in 2 L TSB medium over 50 hours. All strains exhibited slight but consistent increases in conductivity, consistent with ionic metabolite release during growth. Values remained within expected physiological ranges. Each strain was tested in duplicate (1 and 2 represent independent parallel replicates). Ab: *Acinetobacter bohemicus*; Bv: *Bacillus velezensis*; Pc: *Pseudomonas chlororaphis*; Pk: *Pseudomonas kermanshahensis*; Pp: *Pseudomonas protegens*.

Table S4

Microbiological contamination analysis after 2 L scale-up. Results are expressed in CFU/mL or CFU/25 mL. "n.d." indicates "not detected" based on qualitative assays. Ab: *Acinetobacter bohemicus*; Bv: *Bacillus velezensis*; Pc: *Pseudomonas chlororaphis*; Pk: *Pseudomonas kermanshahensis*; Pp: *Pseudomonas protegens*.

**Parameter**	**ISO standard**	**Ab**	**Bv**	**Pc**	**Pk**	**Pp**
Bacillus cereus	ISO 7932	<1 CFU/mL	<1 CFU/mL	<1 CFU/mL	<1 CFU/mL	<1 CFU/mL
Coagulase-positive *Staphylococcus* spp.	ISO 6888	<1 CFU/mL	<1 CFU/mL	<1 CFU/mL	<1 CFU/mL	<1 CFU/mL
Campylobacter spp.	ISO 10272-1	n.d. CFU/25 mL	n.d. CFU/25 mL	n.d. CFU/25 mL	n.d. CFU/25 mL	n.d. CFU/25 mL
Listeria monocytogenes	ISO 11290-1	n.d. CFU/25 mL	n.d. CFU/25 mL	n.d. CFU/25 mL	n.d. CFU/25 mL	n.d. CFU/25 mL
Salmonella spp.	ISO 6579-1	n.d. CFU/25 mL	n.d. CFU/25 mL	n.d. CFU/25 mL	n.d. CFU/25 mL	n.d. CFU/25 mL
E. coli and coliforms	ISO 9308-1	<1 CFU/mL	<1 CFU/mL	<1 CFU/mL	<1 CFU/mL	<1 CFU/mL
Fecal enterococci	ISO 7899-2	<1 CFU/mL	<1 CFU/mL	<1 CFU/mL	<1 CFU/mL	<1 CFU/mL
Pseudomonas aeruginosa	ISO 16266	<1 CFU/mL	<1 CFU/mL	<1 CFU/mL	<1 CFU/mL	<1 CFU/mL

Table S5

Observed standard deviation (%) in measured dioxin concentrations across sampling sites (ERA, SIG, AST) for individual congeners and total toxic equivalent (TEQ) values. Values represent the variability among replicate analyses.

Compound	ERA	SIG	ATS
1.2.3.4.6.7.8-HpCDD	22%	12%	18%
1.2.3.4.6.7.8-HpCDF	11%	14%	22%
1.2.3.4.7.8.9-HpCDF	20%	21%	20%
1.2.3.4.7.8-HxCDD	13%	19%	23%
1.2.3.4.7.8-HxCDF	19%	17%	26%
1.2.3.6.7.8-HxCDD	15%	19%	27%
1.2.3.6.7.8-HxCDF	17%	17%	21%
1.2.3.7.8.9-HxCDD	9%	17%	22%
1.2.3.7.8-PeCDD	15%	18%	20%
1.2.3.7.8-PeCDF	19%	23%	9%
2.3.4.6.7.8-HxCDF	18%	18%	26%
2.3.4.7.8-PeCDF	5%	17%	14%
2.3.7.8-TCDF	24%	35%	20%
OCDD	18%	12%	20%
OCDF	13%	14%	22%
TEQ (WHO-05) incl 0% LQ	12%	18%	20%
TEQ (WHO-05) incl 100% LQ	11%	18%	18%
TEQ (WHO-05) incl 50% LQ	11%	18%	19%

HpCDD: heptachlorodibenzo-p-dioxin; HpCDF: heptachlorodibenzofuran; HxCDD: hexachlorodibenzo-p-dioxin; HxCDF: hexachlorodibenzofuran; PeCDD: pentachlorodibenzo-p-dioxin; PeCDF: pentachlorodibenzofuran; TCDF: tetrachlorodibenzofuran; OCDD: octachlorodibenzo-p-dioxin; OCDF: octachlorodibenzofuran.

Table S6

Relative variation (%) in dioxin and furan congener concentrations following bioremediation treatments at three soil sampling sites (ERA, AST, SIG) after 4, 8, and 24 weeks. Treatment 1 consists of *A. bohemicus* and *B. velezensis*; Treatment 2 includes all five isolated strains. Absolute concentrations for each congener are provided in Table S1.

	AST			AST			ERA			ERA		
	Treatment 1			Treatment 2			Treatment 1			Treatment 2		
Compound	4 weeks	8 weeks	24 weeks	4 weeks	8 weeks	24 weeks	4 weeks	8 weeks	24 weeks	4 weeks	8 weeks	24 weeks
1.2.3.4.6.7.8-HpCDD	93%	110%	165%	116%	119%	140%	136%	78%	114%	92%	100%	89%
1.2.3.4.6.7.8-HpCDF	88%	110%	171%	124%	102%	144%	91%	110%	130%	110%	110%	95%
1.2.3.4.7.8.9-HpCDF	94%	118%	165%	118%	141%	141%	83%	100%	117%	133%	117%	133%
1.2.3.4.7.8-HxCDD	100%	113%	150%	125%	100%	138%	87%	96%	122%	113%	96%	104%
1.2.3.4.7.8-HxCDF	83%	108%	183%	133%	108%	142%	81%	97%	135%	119%	108%	114%
1.2.3.6.7.8-HxCDD	94%	118%	200%	129%	118%	153%	98%	113%	128%	113%	113%	113%
1.2.3.6.7.8-HxCDF	89%	116%	187%	124%	124%	160%	83%	83%	139%	117%	122%	111%
1.2.3.7.8.9-HxCDD	95%	105%	171%	124%	124%	143%	92%	92%	131%	111%	105%	105%
1.2.3.7.8-PeCDD	100%	100%	133%	117%	100%	133%	80%	120%	187%	120%	147%	133%
1.2.3.7.8-PeCDF	105%	126%	189%	105%	105%	126%	74%	89%	148%	119%	133%	104%
2.3.4.6.7.8-HxCDF	85%	111%	170%	136%	102%	136%	79%	91%	139%	115%	109%	109%
2.3.4.7.8-PeCDF	80%	120%	180%	100%	120%	160%	91%	103%	126%	103%	103%	80%
2.3.7.8-TCDF	100%	100%	100%	100%	100%	100%	67%	67%	100%	100%	100%	100%
OCDD	92%	102%	166%	120%	104%	136%	127%	81%	108%	95%	91%	89%
OCDF	88%	123%	180%	127%	105%	136%	89%	99%	137%	116%	109%	92%
TEQ 0% LQ	93%	109%	160%	120%	108%	141%	88%	104%	153%	114%	122%	113%
TEQ 100% LQ	94%	108%	156%	119%	108%	138%	88%	103%	146%	113%	120%	112%
TEQ 50% LQ	93%	108%	158%	119%	108%	139%	88%	103%	150%	113%	121%	113%

HpCDD: heptachlorodibenzo-p-dioxin; HpCDF: heptachlorodibenzofuran; HxCDD: hexachlorodibenzo-p-dioxin; HxCDF: hexachlorodibenzofuran; PeCDD: pentachlorodibenzo-p-dioxin; PeCDF: pentachlorodibenzofuran; TCDF: tetrachlorodibenzofuran; OCDD: octachlorodibenzo-p-dioxin; OCDF: octachlorodibenzofuran.

Data for the SIG site are shown on the next page.

Table S6 (continued)

Compound	SIG			SIG		
	Treatment 1			Treatment 2		
	4 weeks	8 weeks	24 weeks	4 weeks	8 weeks	24 weeks
**1.2.3.4.6.7.8-HpCDD**	109%	45%	80%	114%	97%	93%
**1.2.3.4.6.7.8-HpCDF**	104%	50%	78%	120%	100%	100%
**1.2.3.4.7.8.9-HpCDF**	111%	46%	75%	129%	93%	111%
**1.2.3.4.7.8-HxCDD**	114%	46%	70%	124%	84%	102%
**1.2.3.4.7.8-HxCDF**	103%	48%	79%	126%	96%	112%
**1.2.3.6.7.8-HxCDD**	119%	53%	77%	119%	88%	106%
**1.2.3.6.7.8-HxCDF**	108%	49%	74%	124%	108%	107%
**1.2.3.7.8.9-HxCDD**	114%	43%	75%	120%	92%	102%
**1.2.3.7.8-PeCDD**	111%	46%	63%	124%	78%	92%
**1.2.3.7.8-PeCDF**	125%	52%	69%	121%	85%	89%
**2.3.4.6.7.8-HxCDF**	103%	52%	82%	127%	100%	105%
**2.3.4.7.8-PeCDF**	119%	55%	78%	113%	96%	101%
**2.3.7.8-TCDF**	127%	59%	78%	137%	98%	88%
**OCDD**	108%	47%	83%	115%	90%	105%
**OCDF**	104%	47%	78%	120%	98%	105%
**TEQ 0% LQ**	111%	48%	77%	123%	89%	98%
**TEQ 100% LQ**	111%	48%	77%	123%	89%	98%
**TEQ 50% LQ**	111%	48%	77%	123%	89%	98%

HpCDD: heptachlorodibenzo-p-dioxin; HpCDF: heptachlorodibenzofuran; HxCDD: hexachlorodibenzo-p-dioxin; HxCDF: hexachlorodibenzofuran; PeCDD: pentachlorodibenzo-p-dioxin; PeCDF: pentachlorodibenzofuran; TCDF: tetrachlorodibenzofuran; OCDD: octachlorodibenzo-p-dioxin; OCDF: octachlorodibenzofuran.
